# From introduced American weed to Cape Verde Islands endemic: the case of *Solanum rigidum* Lam. (Solanaceae, *Solanum* subgenus *Leptostemonum*)

**DOI:** 10.3897/phytokeys.25.4692

**Published:** 2013-07-18

**Authors:** Sandra Knapp, Maria S. Vorontsova

**Affiliations:** 1 Department of Life Sciences, Natural History Museum, Cromwell Road, London SW7 5BD; 2 Herbarium, Library, Art and Archives, Royal Botanic Gardens Kew, Richmond, Surrey TW9 3AB

**Keywords:** Africa, aubergine, Cape Verde Islands, Caribbean, description, eggplant, typification

## Abstract

A *Solanum* species long considered an American introduction to the Cape Verde Islands off the west coast of Africa is identified as *Solanum rigidum*, a member of the Eggplant clade of Old World spiny solanums (*Solanum* subgenus *Leptostemonum*) and is probably endemic to the Cape Verde Islands. Collections of this species from the Caribbean are likely to have been introduced from the Cape Verde Islands on slave ships. We discuss the complex nomenclatural history of this plant and provide a detailed description, illustration and distribution map. The preliminary conservation status of *Solanum rigidum* is Least Concern, but needs to be reassessed in light of its endemic rather than introduced status.

## Introduction

*Solanum* L. (Solanaceae) is the largest genus of Solanaceae; with some 1400 species, it is one of the largest angiosperm genera (Frodin 2004). Species of *Solanum* occur on all temperate and tropical continents, with the highest diversity of both groups and species in tropical South America, concentrated in circum-Amazonia (Knapp 2002). The last time *Solanum* was monographed in its entirety was in De Candolle’s *Prodromus* (Dunal 1852), which included 901 species (with an additional 19 incompletely known). *Solanum* taxonomy has proceeded in a piecemeal fashion until relatively recently and the genus has acquired a reputation of being intractable. A project funded by the United States National Science Foundation’s Planetary Biodiversity Inventory (PBI) program begun in 2004 seeks to redress this situation by accelerating species-level taxonomic work across the entire genus. Current work by participants of the “PBI Solanum” project (see www.nhm.ac.uk/solanaceaesource) will result in a modern monographic treatment of the genus available online. This contribution is part of that collaborative effort.

Although *Solanum* is predominantly a New World group, two clades of Old World species are resolved in molecular phylogenetic analyses (Bohs 2005). The largest of these is the “Old World Clade” of the spiny (prickly) solanums (subgenus *Leptostemonum* Bitter) which comprises approximately 100 species across Asia and Africa, plus an additional ca. 100 species endemic to Australia (Symon 1981). We have recently completed a monograph of the spiny solanums of continental Africa and Madagascar (Vorontsova and Knapp in press) and although species from the offshore African islands such as the Canaries and Cape Verdes were not included, their status and morphology were reviewed as part of the larger work. In so doing, we found that a species from the Cape Verde Islands that was considered an introduction from the New World was in fact a member of the Old World Clade of the spiny solanums. In addition, the name by which it had been identified for over 70 years was ambiguous and confusing and was proposed for rejection (Knapp 2011) under the rules of the *International Code of Nomenclature for algae, fungi, and plants* (McNeill et al. 2012). Here we provide a description of this species, ascertain its correct name and discuss its probable relationships.

## Materials and methods

This study is based on examination of herbarium specimens from the herbaria listed in the text, and comparison with a large (>5000) number of specimens of *Solanum* species from the African continent used in the preparation of our monograph on the prickly *Solanum* of continental Africa and Madagascar (Vorontsova and Knapp in press). Type specimens were obtained on loan from the relevant herbaria, or examined digitally through JSTOR Global Plants (http://plants.jstor.org/) and Sonnerat (http://coldb.mnhn.fr/colweb/form.do?model=SONNERAT). We cite all specimens examined that have locality data here, and full details can be found on the Solanaceae Source website (http://www.solanaceaesource.org).

## Taxonomic treatment

### Solanum rigidum

Lam., Tabl. Encycl. 2: 23. 1794.

urn:lsid:ipni.org:names:820779-1

http://species-id.net/wiki/Solanum_rigidum

Figs 1 – 3

Solanum latifolium Lam., Encycl. (Lamarck) 4: 303. 1797. urn:lsid:ipni.org:names:819759-1 Type: Cultivated in the Jardin du Roi in Paris, “Cette plante a été cultivée au jardin des Plantes. Ce soupçonne originaire d’Amerique” (no specimens cited, none found; synonymy ex descr., see Discussion).
Solanum heteracanthum Dunal, Encycl. Suppl. [Poiret] 3: 773. 1814. urn:lsid:ipni.org:names:819434-1 Type: “Africa?”, sin. loc., *DuPuiss.n.* (lectotype, designated here: P [P00344411]).
Solanum rigidum Type: Cultivated in the Jardin du Roi in Paris, origin unknown, *Anon.s.n.* (lectotype, designated here: P [P00357615]).

#### Description.

Herbaceous subshrub to shrub, 0.6–1.5 m tall, armed; stems erect, densely (occasionally sparsely) pubescent with sessile or short-stalked translucent stellate trichomes < 0.5 mm long, the rays 4–6, ca. 0.5 mm long, the midpoints equal to the rays, prickly with straight to slightly curved broad-based prickles of varying lengths, 2–6 mm long, these sparsely stellate-pubescent; new growth densely stellate-pubescent, yellowish-brown in dry plants; bark of older stems dark greyish brown, not markedly glabrescent. Sympodial units difoliate and usually geminate, the leaves of a pair equal in size and shape. Leaves simple, 4.5–14(18) × 3.5–8.5(14) cm, elliptic, membranous to somewhat chartaceous, concolorous; upper surfaces sparsely pubescent with sessile (with some short-stalked) translucent stellate trichomes, the rays 3–4, ca. 0.3 mm long, the midpoint equal to the rays or occasionally somewhat longer; lower surfaces more densely pubescent with short-stalked or occasionally sessile translucent stellate trichomes to 0.3 mm long, the rays 4–5(-6), to 0.6 mm long, the midpoint equal to the rays, the lamina still easily visible; primary veins 3–5 pairs, drying yellowish, with 2–6 pale tan prickles to 6 mm long on both surfaces; base attenuate, usually decurrent onto the petiole; margins shallowly lobed, the lobes 3–4 on each side of the midrib, lobed 1/4–1/3 of the way to the midrib, the apices acute; apex acute; petioles usually somewhat winged from the decurrent leaf bases, 1.5–4.5 cm long, densely stellate-pubescent like the stems, usually with a few pale tan prickles of varying lengths, to 6 mm long, these straight or very slightly curved. Inflorescence 1–2.5 cm long, internodal (lateral) or opposite the leaves, simple or only once branched, with 5–6(10) flowers, the lowermost flower(s) hermaphroditic and the plants andromonoecious, densely pubescent with sessile or short-stalked translucent stellate trichomes with rays to 0.3 mm long like those of the stems; peduncle (0-)1–2 cm long, the lowermost flower often borne at the very base of the inflorescence; pedicels 1–1.5 cm long, 1–2 mm in diameter, densely stellate-pubescent like the rest of the inflorescence, that of the lowermost hermaphroditic flower usually somewhat stouter and usually with >10 pale tan prickles to 4 mm long, usually nodding at anthesis, articulated at the base; pedicel scars unevenly spaced 1–5 mm apart, the distance greatest between the lowermost flower and the rest. Buds elongate ellipsoid, the corolla approximately halfway exserted from the calyx before anthesis. Flowers 5-merous, strongly heterostylous, the lowermost (or lowermost 2–3) flower long-styled and hermaphroditic, usually slightly larger than the rest, the distal flowers short-styled and functionally male. Calyx tube 4–4.5 mm long, cup-shaped, the lobes 6–7 mm long, narrowly triangular with attenuate tips, densely pubescent with sessile and short-stalked stellate trichomes like those of the rest of the inflorescence, those of the hermaphroditic (long-styled) flowers usually with 5–25 yellowish prickles to 4 mm long. Corolla 2.5–3 cm in diameter, violet or white (type only), pentagonal with abundant interpetalar tissue, lobed less than 1/4 of the way to the base, the lobes 2–5 × 2–5 mm, usually with small acumens, planar at anthesis, glabrous adaxially, occasionally with a few stellate trichomes on the tips and margins, densely stellate-pubescent abaxially in a band 1.5 mm wide on either side of the petal midvein (exposed area in bud), the interpetalar tissue glabrous and thinner. Stamens equal, the filament tube ca. 0.5 mm long, the free portion of the filaments ca. 1.5 mm long, glabrous; anthers 6–7 × ca. 1.5 mm, usually slightly longer in hermaphroditic flowers, tapering, yellow, poricidal at the tips, the pores directed distally. Ovary densely pubescent with translucent stellate trichomes with 3–5 rays ca. 0.25 mm long; style 12–15 mm long in long-styled flowers, 1–1.5 mm long in short-styled flowers, sparsely pubescent with stellate trichomes like those of the ovary, these denser near the base; stigma expanded, bi-lobed. Fruit a globose berry, 2.5–3.5 cm in diameter, usually only 1 per infructescence, when immature mottled green and whitish green, maturing yellow, the pericarp leathery, ca. 1 mm thick, glabrous when mature; fruiting pedicels 2–2.5 cm long, 2–3 mm in diameter, thick and woody, pendent, pubescent and prickly as in the flowering pedicels; fruiting calyx splitting to the base, the lobes to 1 cm long, the tips usually reflexed at fruit maturity. Seeds > 100 per berry, 3–4 × 2.5–3 mm, reniform and slightly ovoid, not thickened at the margins, pale yellow or yellowish tan, the testal cells sinuate in outline. Chromosome number not known.

**Figure 1.**
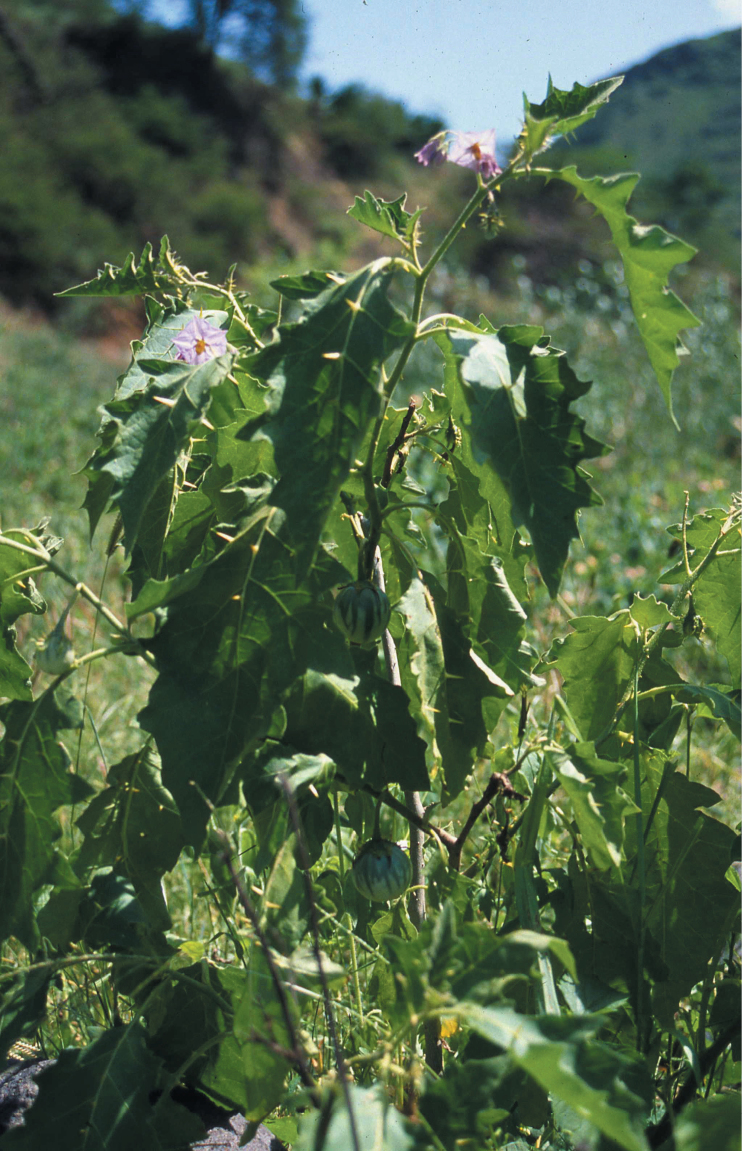
Habit of *Solanum rigidum* on the Cape Verde Islands. Photograph courtesy of MC Duarte.

**Figure 2.**
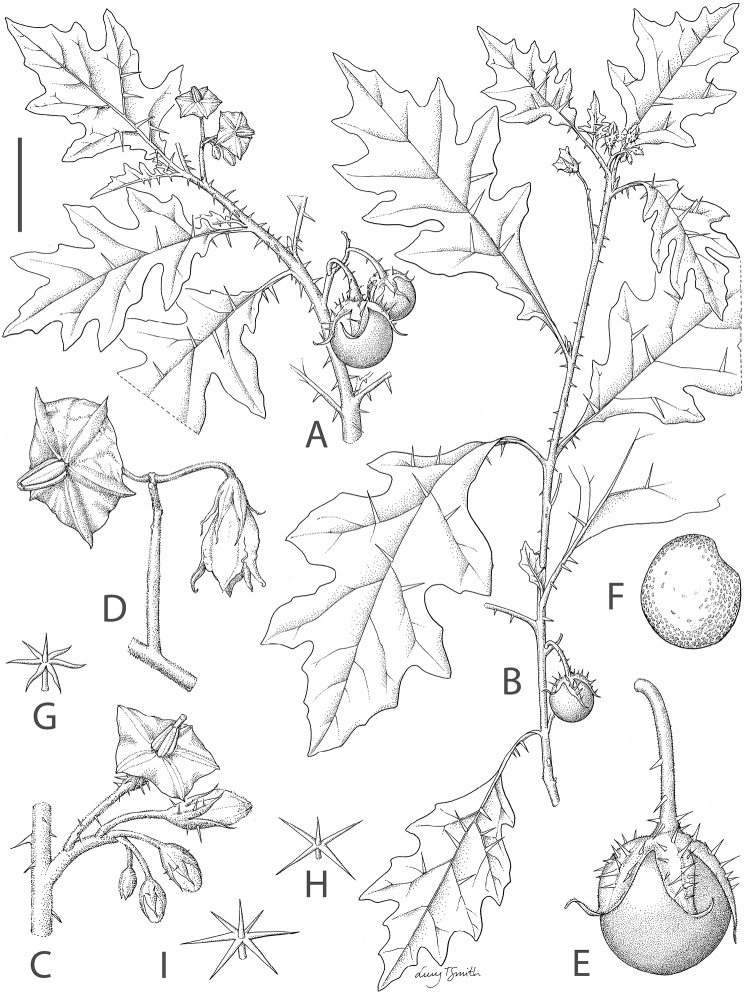
*Solanum rigidum*. **A** habit with dense prickles and small deeply lobed leaves **B** habit with sparse prickles and larger more shallowly lobed leaves **C** inflorescence with a single lowermost hermaphroditic long-styled flower at the base **D** inflorescence with distal functionally male short-styled flowers **E** immature fruit and fruiting calyx with attenuate calyx lobe tips **F** seed **G** stellate trichome from the stem **H, I** stellate trichomes from the lower surface of the leaf. **A**, **C**, **G–I** drawn from *Martins et al 468* **B, F** drawn from *Barbosa & Silva 14072* **D, E** drawn from photographs of MC Duarte. Drawn by Lucy T. Smith.

**Figure 3.**
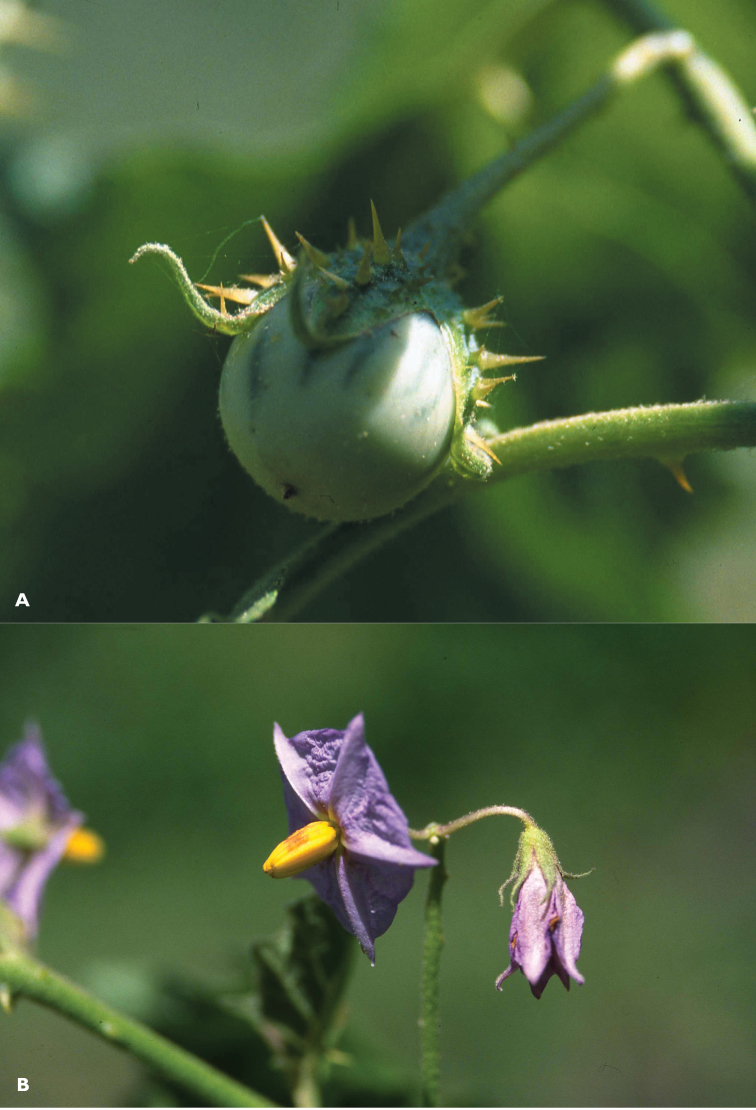
*Solanum rigidum* on the Cape Verde Islands. **A** immature fruit with upturned sepal lobes, **B** functionally staminate flower with copious interpetalar tissue. Photographs courtesy of MC Duarte.

#### Distribution.

**Figure 4.**
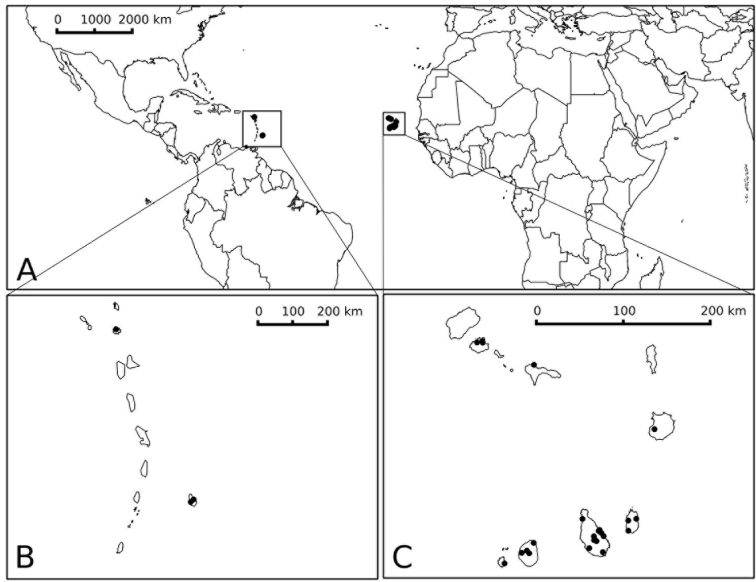
Distribution map of *Solanum rigidum*. **A** global distribution in the Caribbean and the Cape Verdes **B** distribution on Antigua and Barbados **C** distribution on the Cape Verde Islands. Prepared by Paweł Ficinski.

(Fig. 4) Endemic to the Cape Verde Islands, known from seven of the ten islands of the archipelago, on both the windward and leeward arcs; a few old collections from the Caribbean (see Discussion). Like most prickly solanum species, *Solanum rigidum* is a plant of disturbed and open areas and is somewhat weedy; it grows from sea level to 100 m.

#### Common names and uses.

Olho de vaca; olho de boi (cow’s eye, bull’s eye – perhaps in reference to the globose fruits).

#### Discussion.

*Solanum rigidum* has long been treated as *Solanum fuscatum* L. (Linnaeus 1762), and as an American introduction to the Cape Verde Islands, rather than the endemic species that it is. Use of the name *Solanum fuscatum* (proposed for rejection due to its inconsistent and complex usage since its first description, Knapp 2011; rejection supported by Committee on Vascular Plants [17 for, 0 against, 1 abstention], see Applequist 2012) began in the early 20^th^ century with Chevalier’s (1935: 904) treatment of the plants of the Cape Verde archipelago; he assumed that this spiny solanum species was an introduction from the Americas, presumably from Linnaeus’ indication of origin of *Solanum fuscatum*, or Dunal’s (1814) suggestion that his *Solanum heteracanthum* was similar to “*Solanum fuscatum*”, and that assumption has persisted in floristic work since that time.

We have lectotypifed *Solanum rigidum* with a sheet in the Lamarck herbarium (P00357615, online at http://tinyurl.com/rigidum-LT) that is annotated with a reference to “illustr.”, the original place of publication (Lamarck 1795). *Solanum latifolium* was also described by Lamarck (1797) a few years after *Solanum rigidum*; he stated “Cette espèce a de très-grandes rapports avec le precedente [S. rigidum], dont elle differe por beaucoup moins de roideur, por sa tige droite, beaucoup plus elevée, por de très grandes feuilles large, & por ses grappes de fleur disposées le long de branches, & moins terminals” [This species has great affinity with the preceding, but differs in that it is less rigid, its straight stem that is much taller, its large leaves and its inflorescences disposed along the branches and less terminal]. We have not found specimens that match the protologue, but have not yet exhaustively searched the herbarium at P, where such a sheet, if it exists, should be found; we therefore include this name in synonymy from the description, and postpone lectotypification until an exhaustive search has been made. *Solanum latifolium* has been attributed to Abbé Jean Louis Marie Poiret by some authors (e.g., Gooding 1965), and many specimens of the Cuban endemic *Solanum gundlachii* Urb. (see Knapp 2009) were annotated as such. The epithet *latifolium* clearly is attributable to Lamarck; Poiret was responsible for volumes 5–8 of the *Encyclopedie Methodique*, not volume 4 in which the epithet appears. A fragment of leaf at F (F-676645) labelled “Solanum latifolium Poir.” in Macbride’s hand with a stamp indicting it originated from P matches the pubescence and prickliness of *Solanum rigidum*, but there are no flowers or fruits or leaf bases to be certain of its identity.

In describing *Solanum heteracanthum* Dunal (1814) cited a specimen in the Richard herbarium (“Dunal, in herb. Rich.”) now held at P (P00344411). This is likely to be the holotype specimen but we prefer to designate this the lectotype as no specific sheet nor collector were cited in the protologue. This sheet is of a particularly prickly and possibly juvenile plant of *Solanum rigidum*; it has the characteristic elongate calyx lobes of this species.

A few collections from the Caribbean have confused understanding of the origins and distribution of this species; these plants may represent early introductions via the transatlantic routes used to transport the enslaved from Africa to the New World; these routes all passed through the Cape Verde archipelago from about 1560. Gooding et al. (1965) included the species (as *Solanum latifolium* Poir.) in their *Flora of Barbados*, and recorded its presence on Antigua and Barbados. We have seen no material referable to *Solanum rigidum* from later than 1935 nor collections from elsewhere in the West Indies (Knapp 2012); if it were a widespread introduction from the Caribbean we would expect collections from the Windward Islands, as most *Solanum* species from that region are found on multiple islands. Some records of “*Solanum latifolium*” may be prickly individuals of the cultivated eggplant, *Solanum melongena* L.; careful examination of specimens is necessary to determine the status of these records. Other members of the Eggplant clade are used medicinally in Africa and Asia (Meyer et al. 2011; Vorontsova and Knapp 2012) and this may account for the occurrence of *Solanum rigidum* in the Caribbean.

Morphologically, *Solanum rigidum* does not resemble any American species or species group of solanums, but it is more similar to African species of the Eggplant clade such as *Solanum cerasiferum* Dunal and *Solanum campylacanthum* A.Rich. in its greyish green leaves (Fig. 1), violet flowers and spherical fruits with tough pericarp that is green-striped when immature (see Fig. 3A) and yellow when ripe. *Solanum rigidum* differs from those species in its densely pubescent ovaries and long acuminate calyx lobes that are upturned in fruit (Fig. 3A). It shares with those taxa a strongly andromonoecious breeding system, with a single or few hermaphroditic flowers at the base of the inflorescence and the distal flowers with short styles and functioning as males (Fig. 3B). Andromonoecy is common in the spiny solanums (subgenus *Leptostemonum* Bitter) and is found in both New and Old World species.

Two other *Solanum* species occur on the Atlantic islands off the African coast (Macronesia): *Solanum verspertilio* Aiton and *Solanum lidii* Sunding, both from the Canary Islands. Both those species have strongly zygomorphic flowers with strongly unequal anthers while the flowers of *Solanum rigidum* are actinomorphic with anthers of equal size.

Preliminary DNA sequence data (S. Stern and M.S. Vorontsova pers. comm.) also indicate that *Solanum rigidum* is a member of the Eggplant clade, a large group of mostly East African taxa that includes the cultivated eggplant *Solanum melongena* L. *Solanum rigidum* may be of hybrid origin; in preliminary plastid analyses it is sister to *Solanum campylacanthum* while in trees based on the nuclear ITS region it is sister to *Solanum macrocarpon* L. (the gboma eggplant, a continental African species). The chromosome number of *Solanum rigidum* is not known, but *Solanum campylacanthum* is tetraploid in some parts of its range (see Knapp et al. 2013), while *Solanum macrocarpon* is diploid. The preliminary relationships based on molecular data may indicate hybrid origin, or introgression; further work on the cyotogenetics of all members of the Eggplant clade is a priority (see Knapp et al. 2013).

The Cape Verde islands are geologically linked with the Canary Island archipelago (Patriat and Labails 2006) and have similar histories with the main volcanic episodes resulting in island emergence in the Cenozoic, although the basement igneous rocks are much older. The endemic *Solanum* species of the Canary Islands, *Solanum vespertilio* Aiton and *Solanum lidii* Sunding, are not closely related to the Eggplant clade but to South African taxa (Anderson et al. 2006) such as *Solanum capense* L. or to the paraphyletic “Anguivi grade” (Vorontsova et al. 2013). This supports a scenario where *Solanum rigidum* is the result of dispersal from the African mainland to the Cape Verdes, a distance of only 570 kilometres. Further molecular work, however, will be necessary to understand its origins and detailed relationships.

The discovery that *Solanum rigidum* is not an introduction from the Americas but instead an endemic species in the Cape Verde islands highlights the need for conservation assessment on the islands in order to determine its range and population sizes. *Solanum rigidum* occurs on both of the main island groups of the Cape Verdes, on São Vincente, São Nicolau and Boa Vista of the Ilheus de Barlovento, and on Maio, Santiago, Fogo and Brava of the southern Ilheus de Sotovento (Fig. 4). Label data indicate *Solanum rigidum* occurs in disturbed habitats, often at the edges of washes and riverbeds, so it may be a weedy species despite its narrow geographic range and endemic status. Applying the IUCN criteria (IUCN 2001) results in a preliminary conservation status of Least Concern, given its occurrence on many of the islands of the archipelago, but given the endemic status of *Solanum rigidum* this should be re-assessed in the light of more accurate population and threat status levels with better field data.

#### Specimens examined.

**Cape Verde Islands.** **Boa Vista**: sin. loc., 7 July 1934, *A. Chevalier 44897* (P). **Brava**: Cachaço, Cova do Mar, 200 m, 29 October 1983, *G. Cardoso de Matos 5434* (LISC); on the Ponton Road to the Fort, 26 March 1864, *R.T. Lowe* *s.n.* (P); **Fogo**: entre as povoações de Lomba e Ribeira Filipe, 900 m, 1 November 1983, *G. Cardoso de Matos 5505* (LISC); San Filipe, 18 July 1934, *A. Chevalier 44800* (P); Mosteiros, junto a pista de aviação, 10 m, 13 October 1991, *Martins 468* (LISC); Curral da Chão, entre Achada Furna e Miguel Gonçalves, 15 October 1991, *Martins 510* (LISC). **Maio**: Pedro Vaz, 17 May 1956, *L.A. Grandvaux Barbosa 7437* (LISC); Calheta, 9 November 1964, *J. Malato-Beliz 141* (LISC); Pedro Vaz, 11 November 1964, *J. Malato-Beliz 244* (LISC); Vila da Maia, Dunas de Morrinho, 17 November 1964, *J. Malato-Beliz 360* (LISC). **Santiago**: São Jorge dos Orgãos, Ribeirão Galinha, 350 m, 22 October 1983, *G. Cardoso de Matos 5304* (LISC); Ribeira Grande de Santiago, a longo de leito seco da Ribeira de Fundão depois de passar os regadios que estão junto as casas, 540 m, 15 July 1993, *M.C. Duarte 540* (LISC); estrada Praia-Tarrafal, 1.1 km depois Porto Fundo, 65 m, 9 December 1955, *L.A. Grandvaux Barbosa 5909* (LISC); Santa Cruz, aluviões do Ribeira da Cruz, 16 November 1982, *L.A. Grandvaux Barbosa 14072* (LISC); Ribeira de Santa Cruz, 20 March 1983, *L.A. Grandvaux Barbosa 14486* (LISC); between S. Domingo and Os Orgãos, 31 January 1866, *R.T. Lowe* *s.n.* (BM, LE); Villa do Praia, January 1861, *F.M.J. Welwitsch 6086* (BM); Pedra Badejo, 10 m, 17 October 1992, *M.C. Duarte 56* (LISC); Chã de Vaca, 277 m, 16 October 1994, M.C. Duarte 701 (LISC); Foz da Ribeira de Mangue, 30 m, 11 July 1993, *M.C. Duarte 502* (LISC); Baia de Chão Bom, 2 m, 23 October 1994, *M.C. Duarte 1189a* (LISC); sin. loc, *C. Peters* *s.n.* (LE). **São Nicolau**: am Weg von Estancia Bras zum Ribeira Quameros, 50 m, 3 January 1986, *N. Kilian 1014* (B). **São Vicente**: Monte Verde, September 1934, *A. Chevalier 45744* (P); Porto Grande, 22 November 1894, *E.H.L. Krause 17687* (B); ascent of Monte Verde, 609 m, 5 January 1866, *R.T. Lowe* *s.n.* (BM).

**Antigua and Barbuda.** **Antigua**: sin. loc, *Anonymous 16* (K).

**Barbados.** **Barbados**: Foster Hall Spring, January 1890, *H.F.A. Eggers 7226* (P); near Welches, St. Thomas, June 1935, *A.C.S. McIntosh 195* (P); Bathsheba and Hastings, April 1895, *J.F. Waby 53a* (BM, K).

## Supplementary Material

XML Treatment for Solanum rigidum
